# Immunomodulatory and immunotherapeutic implications of tobacco smoking in squamous cell carcinomas and normal airway epithelium

**DOI:** 10.18632/oncotarget.26982

**Published:** 2019-06-11

**Authors:** Jingming Wang, Maximilian Linxweiler, Wei Yang, Timothy A. Chan, Luc G.T. Morris

**Affiliations:** ^1^ Immunogenomics Precision Oncology Platform, Memorial Sloan Kettering Cancer Center, New York, NY, USA; ^2^ Human Oncology and Pathogenesis Program, Memorial Sloan Kettering Cancer Center, New York, NY, USA; ^3^ Department of Radiation Oncology, Memorial Sloan Kettering Cancer Center, New York, NY, USA; ^4^ Department of Surgery, Memorial Sloan Kettering Cancer Center, New York, NY, USA

**Keywords:** immune, microenvironment

## Abstract

The mutagenic effects of tobacco smoking increase the risk of the development of cancers of the lung, head and neck, and other anatomic sites. In a comparison of squamous cell carcinomas of the lung and the head and neck, we find that the immunomodulatory effects of smoking differ based on anatomic site. In both sites, the mutational signature of smoking is strongly associated with somatic mutational load. In head and neck squamous cell carcinoma, the mutational signature of tobacco exposure is associated with a strongly immunosuppressive tumor microenvironment. In contrast, in lung squamous cell carcinoma, the opposite effect is seen, with the tumor immune microenvironment significantly more inflamed. These effects are mirrored in rates of response to immune checkpoint inhibitor immunotherapy, which tend to be higher in smokers with lung cancer, but lower in smokers with head and neck cancer. We find a similarly strong immunosuppressive effect of smoking in non-cancerous lung epithelium. Taken together, our findings show that the effects of mutational signatures on the immune microenvironment and response to immunotherapy can be affected by context such as cancer type, anatomic site, and histology.

## INTRODUCTION

Traditionally, cancers have been categorized by features such as anatomic site and tissue histology. Our understanding of cancer biology has subsequently evolved to recognize that tumors arising from diverse anatomic sites may share oncogenic molecular drivers or biomarkers. In recent years, targeted and immunotherapeutic drugs have demonstrated activity across multiple cancer types that are defined by a shared oncogenic alteration, such as a driver gene or fusion, or a shared biological process, such as DNA mismatch repair deficiency. This has led some to speculate that oncology drug treatments may be effective, and can receive regulatory approval, based only on molecular markers, in a “tissue-agnostic” fashion. It remains unknown how broadly tissue-agnostic approaches are applicable, and whether context such as anatomic site or tissue histology may alter the implications of these molecular markers [1–3].

Tobacco smoking contributes to the development of multiple cancers, including head and neck and lung cancer [4, 5]. Tobacco smoke contains many carcinogenic chemicals that disrupt DNA such as polycyclic aromatic hydrocarbons and nitrosamines, which can cause G→T transversions and distinct mutational signatures [6, 7]. Apart from increasing the somatic mutational burden associated with unrepaired DNA damage, tobacco carcinogens also appear to alter both the innate and the adaptive immune system, which may contribute to tumorigenesis [8–10]. The likelihood of a tumor responding to immunotherapy treatments such as immune checkpoint blockade (ICB) is known to be affected by these same factors, namely tumor mutational burden (TMB) and the degree of immune infiltration in the tumor microenvironment [11–15].

Immune checkpoint inhibitors are a class of cancer immunotherapy drugs that seek to inhibit T-cell checkpoints [16]. Pivotal trials have shown that a subset of patients with advanced melanoma, non-small cell lung cancer (NSCLC), renal cell carcinoma (RCC), bladder cancer, head and neck squamous cell carcinoma (HNSC), and many other tumor types, experience clinical responses following ICB therapy [17–23]. However, there are still a large majority of patients who do not experience tumor response to ICB, with response rates of 10–20% in HNSC and NSCLC. Interestingly, early clinical observations demonstrated that lung cancer patients with a smoking history tended to have a higher likelihood of ICB response; however, for HNSC patients, the likelihood of response appeared to be lower in smokers [4, 5, 11, 18, 19].

In order to understand how tobacco smoking affects the tumor immune microenvironment and potentially identify the biomarkers to guide treatment options, we analyzed RNA and DNA sequencing data from cases studied as part of The Cancer Genome Atlas (TCGA), as well as two independent gene expression datasets of lung squamous cell carcinoma (LUSC) and HNSC tumors [4]. We found that the mutational signature of tobacco smoking was evident in both lung and head and neck tumors. In both LUSC and HNSC, we found that a higher mutational smoking signature was associated with a higher TMB, as would be expected.

We then examined the association between the mutational signature of smoking and RNA sequencing-derived measures of tumor immune infiltration and T cell activation. In LUSC, a higher mutational smoking signature was positively associated with levels of immune infiltration, cytolytic activity and interferon-γ pathway signaling, indicating that smoking was associated with a more inflamed tumor immune microenvironment. In HNSC, these associations were all strongly in the opposite direction.

These findings have been apparent from clinical data. It was first observed by the Chan group that the genetic smoking signature was associated with a higher TMB and a higher response to anti-PD-1 immunotherapy [11]. It is possible that this higher response rate was also, in part, driven by a more T cell inflamed microenvironment in patients with a heavy smoking history. In HNSC, the reverse trend appears to emerge – we found that HNSC patients with a clinical smoking history tended to have a lower likelihood benefit from anti-PD-1 immunotherapy. These results underscore that contextual factors such as cancer type and anatomic site may play a role in modulating the interaction between the tumor genome and anti-tumor immunity.

**Figure 1:**
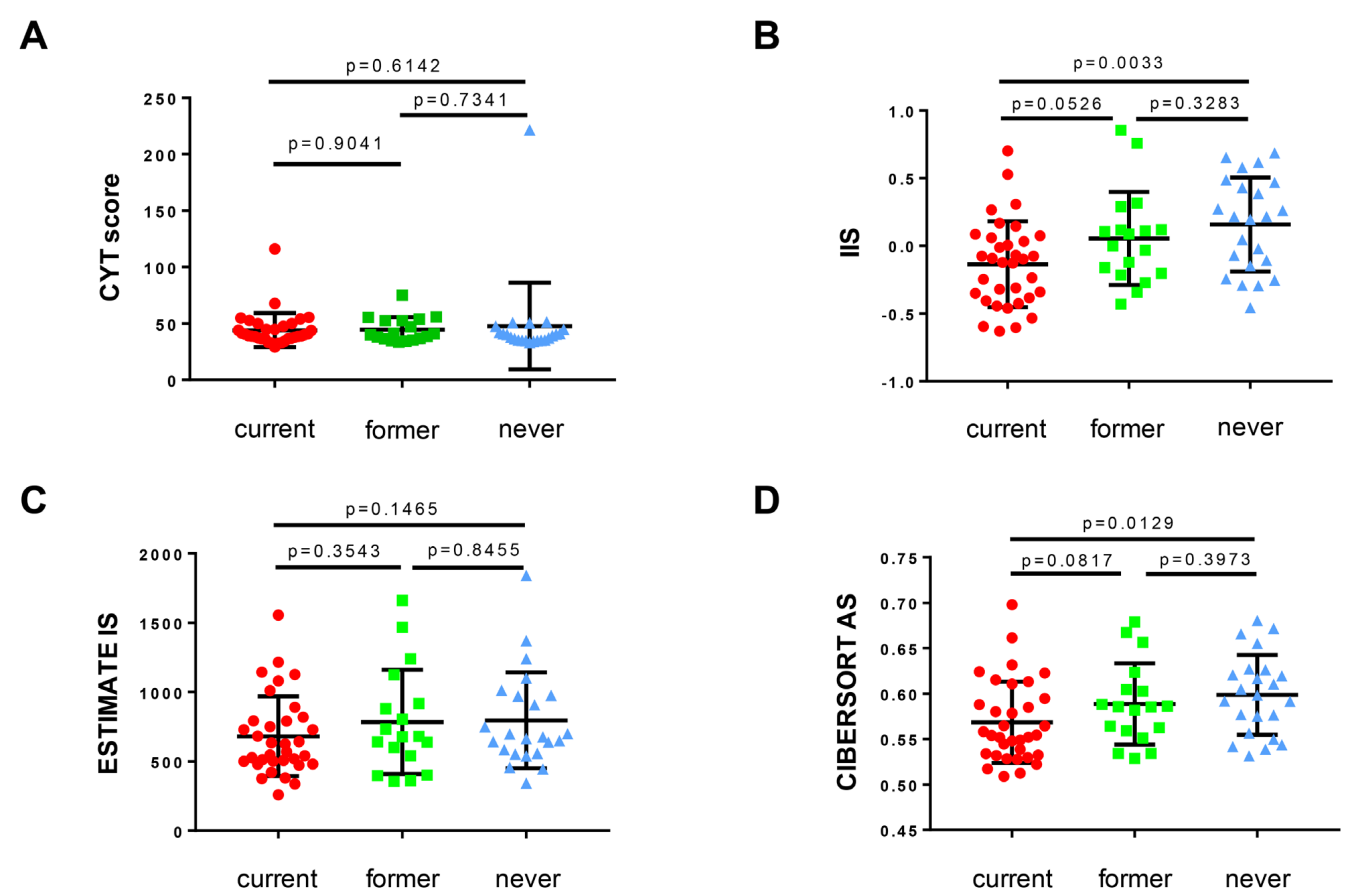
The association of smoking history with immune infiltration and T cell activation in non-cancerous bronchial epithelium demonstrates stepwise immunosuppression with increasing smoking history. Human bronchial mucosa samples (total *n* = 75, current smoker *n* = 34, former smoker *n* = 18, never smoker *n* = 23; clinical and expression data from [24]); (**A**) CYT score, (**B**) IIS, (**C**) ESTIMATE IS, and (**D**) CIBERSORT AS illustrated as scatter blots for current, former and never smokers. For all statistical analyses, a Mann-Whitney-*U* test was used (α = 0.05) and *p*-values ≤ 0.05 were considered statistically significant. CYT score – cytolytic activity score; IIS – immune infiltration score; ESTIMATE IS – ESTIMATE signature score; CIBERSORT AS – CIBERSORT absolute score; current – current smoker (red dots); former – former smoker (green squares); never – never smoker (blue triangles).

Tobacco smoking has both immunosuppressive effects, such as a pro-apoptotic effect on T cells, as well as inflammatory effects. The balance of these sequelae may differ in the epithelium of the lung and bronchus versus upper aerodigestive tract mucosa. Furthermore, they may differ in time – one effect may predominate in normal epithelium, prior to cancer initiation, and the other effect may be more relevant during the development of an early-stage cancer. To better understand the effects of tobacco smoke in sculpting the immune microenvironment of non-cancerous normal cells, we analyzed gene expression data from normal human airway epithelial cells in volunteers undergoing bronchoscopy [24]. In this study, Spira and colleagues obtained bronchoscopic biopsies of normal lower airway epithelium from a group of 34 current smokers, 18 former smokers and 23 never smokers. We analyzed these data and deconvolved gene expression data to measure immune infiltration and T cell activation [25, 26]. We determined levels of immune activation and infiltration by calculating the cytolytic (CYT) score (incorporating cytolytic effectors of CD8+ T cells: *GZMA*, and *PRF1*) [27], immune infiltration score (IIS) [28], ESTIMATE signature score [29] and CIBERSORT absolute score [30] for each sample (Figure 1). In these samples, we observed that smoking was associated with a significantly immunosuppressive microenvironment in normal human airway epithelium, most notably in current smokers, and less so in former smokers.

These findings will require further mechanistic investigation, perhaps with *in vivo* immunocompetent models of smoking carcinogenesis [31, 32]. We believe that these findings are consistent with profoundly immunosuppressive effects of tobacco smoke on the local immune microenvironment, which together with the mutagenic effects of tobacco, can lead to the initiation of cancer. In certain sites or anatomic locations such as the lung, the higher tumor mutational load associated with smoking may lead to an enhancement of T cell infiltration that becomes evident after tumor initiation. This effect is not seen in the head and neck mucosa, possibly because the immunosuppressive effects of smoking are more profound, or the degree of T cell infiltration responding to elevated mutational load is less marked. These effects would then potentially affect the probability of response to immunotherapies [33]. It is important to note that this latter point at present speculative and that the mechanistic aspects of carcinogenesis are likely to be far more complex. Indeed, there are several different carcinogenic compounds in cigarette smoke which are likely to exert differing immunosuppressive and inflammatory effects in different parts of the upper (HNSC) or lower (LUSC) airways.

The effect of smoking on cancer immunity and immunotherapy response needs to be explored across cancer types more broadly. A recent study of melanoma patients found that smoking had a strong negative prognostic effect in highly immune infiltrated tumors [34]. The systemic effects of tobacco smoke on smoking-associated cancers that are not directly exposed to smoke, such as bladder cancer, will also be important to dissect further. Future mechanistic and clinical studies will be needed to elucidate the importance of this risk factor, and its associated mutational signature, to the shaping of the tumor immune microenvironment, and the development of response and resistance to immunotherapies. These data indicate that it is very likely that cancer-causing processes, and mutational signatures, exert different effects on anti-tumor immunity in different contexts, and that further research will need to consider the interaction between molecular carcinogenesis and cancer type/location. Together with TMB, PD-L1 staining, measures of immune infiltration, HLA status, and other factors, it is likely that smoking history and/or the smoking mutational signature will add predictive value to our efforts to define biomarkers of response to ICB.
